# Supplementary material for: Remote sensing techniques for automated marine mammals detection: a review of methods and current challenges
Source: PeerJ. 2022 Jun 20;10:e13540. doi: 10.7717/peerj.13540 (PMC9220915; doi:10.7717/peerj.13540)
Supplement: Table S1 — Number of true positives (TP), false positives (FP) and false negatives (FN) used in our calculations for Equations 3, 4 and 5 in Table 1, together with the source in each study from which they were extracted directly or calculated from. [file peerj-10-13540-s001.docx]

## Supplemental information

**Table S1:**

**Sources for accuracy assessment metrics calculations.**

Number of true positives (TP), false positives (FP) and false negatives (FN) used in our calculations for Equations 3, 4 and 5 in Table 1, together with the source in each study from which they were extracted directly or calculated from.

| Study | Model | TP | FP | FN | Source in study |
| --- | --- | --- | --- | --- | --- |
| Fretwell, Staniland & Forcada (2014) | Unsupervised classification kmeans | 53 | 49 | 38 | Table 1 (Best variants within methods chosen based on Equation 5 for total signals, not by probable, possible and band 5 manual detections) |
|  | Histogram thresholding Band 5 | 77 | 24 | 14 |  |
| Seymour *et al.* (2017) | Saddle Island  (Simple) Pups | 566 | 82 | 39 | Table 3 (only models from prediction site) |
|  | Saddle Island  (Complex) Pups | 515 | 77 | 39 |  |
|  | Saddle Island  (Simple) Adults | 199 | 47 | 2 |  |
|  | Saddle Island  (Complex) Adults | 202 | 100 | 2 |  |
| Thums *et al.* (2018) | Shape algorithm | 71-2 (objects not detected by algorithm that were concluded not to be whales) | 142 | 0 | Tables 2 and 3 and text (p. 32). Calves and mothers together, all images combined |
| Borowicz *et al.* (2019) | CNN (ResNet-152) | 32 | 87 | 0 | Figure 4 (best model chosen by authors) |
| Cubaynes (2019) | Unsupervised classification (Isodata) | 38 | 124 | 50 | Table 4.2 (from total column, mixing definite, probable and possible) |
|  | Supervised (maximum likelihood) | 80 | 3 | 8 |  |
|  | Thresholding (NIR1) | 58 | 171 | 30 |  |
|  | OBIA | 64 | 399 | 24 |  |
| Guirado *et al.* (2019) | Detection CNN | 54 | 5 | 14 | Table S1) A) and B) (supplementary tables) (all locations totals) |
|  | Count CNN | 62 | 3 | 8 |  |
| Gray *et al.* (2019) | CNN | 58 | 1 | 0 | Text (pp. 1495-1496) (only for whale recognition, not species) |
| Gonçalves, Spitzbart & Lynch (2020) | SealNet CNN | 353 | 604 | 815 | Text (pp. 6-7) (total for all scenes) (only model with best F1 for testing) |
